# Shared Control of an Electric Wheelchair Considering Physical Functions and Driving Motivation

**DOI:** 10.3390/ijerph17155502

**Published:** 2020-07-30

**Authors:** Lele Xi, Motoki Shino

**Affiliations:** Department of Human and Engineered Environmental Studies, Graduate School of Frontier Sciences, The University of Tokyo (UT), Chiba 277-8561, Japan; motoki@k.u-tokyo.ac.jp

**Keywords:** electric wheelchair, physical impairments, shared control, driving motivation, reinforcement learning, user-machine interaction

## Abstract

Individuals with severe physical impairments have difficulties operating electric wheelchairs (EWs), especially in situations where fine steering abilities are required. Automatic driving partly solves the problem, although excessive reliance on automatic driving is not conducive to maintaining their residual physical functions and may cause more serious diseases in the future. The objective of this study was to develop a shared control system that can be adapted to different environments by completely utilizing the operating ability of the user while maintaining the motivation of the user to drive. The operating characteristics of individuals with severe physical impairments were first analyzed to understand their difficulties when operating EWs. Subsequently, a novel reinforcement learning-based shared control method was proposed to adjust the control weight between the user and the machine to meet the requirements of fully exploiting the operating abilities of the users while assisting them when necessary. Experimental results showed that the proposed shared control system gradually adjusted the control weights between the user and the machine, providing safe operation of the EW while ensuring full use of the control signals from the user. It was also found that the shared control results were deeply affected by the types of users.

## 1. Introduction

Recently, the number of individuals with mobility problems has increased in Japan [1]. Moreover, maintaining mobility is one of the most important prerequisites for improving quality of life (QOL) [2,3]. Many assistive devices, such as electric wheelchairs (EWs), exoskeletal robotics, and assistive walkers have been developed to help individuals with mobility problems. For severely physically impaired people, their physical functions are extremely weak and only a few muscles can be used to operate assistive robots; therefore, EWs are almost their only tool for maintaining mobility.

With the development of control technologies, sensors, and artificial intelligence technologies, EWs with intelligent controllers have become a growing trend in recent years. With these new technologies, automatic driving and shared control EWs have been developed to help individuals who are unable to operate regular EWs regain their mobility. These state-of-the-art technologies mainly include intention estimation [4], path planning [5], controller design [6], and environmental sensing [7].

Individuals with severe physical impairments usually experience difficulties operating EWs, especially in situations where precise control is necessary, such as approaching an obstacle or turning in a corridor. Automatic driving can solve this problem; however, excessive reliance on automatic driving does not help maintain their residual physical functions and may cause more serious illnesses in the future [8].

Shared control offers the possibility for the user to work together with the machine. Shared control is typically defined as “If the function of the machine augments or replaces one or more of the human senses, the user must be able to reach his goal and also understand what the machine is doing. This two-way interaction must exist in any shared control system” [9].

As discussed above, the use of physical functions is important to maintain the residual physical functions of individuals with severe physical impairments. However, their extremely weak physical functions limit their operating ability. They cannot properly drive EWs without proper assistance, especially in situations where precise control and steering abilities are required. However, too much assistance usually leads them to rely on the system and not to actively try to operate the EW, generating a lazy signal [10]. Therefore, the objective of this study was to develop a shared control system that can be adapted to different environments by completely utilizing the operating ability of users and maintaining their driving motivation. Specifically, work related to shared control for EWs is introduced in Section 2, and the operating characteristics of individuals who have difficulties in operating EWs, along with the requirements of the shared control system, are discussed in Section 3. The construction of the shared control system is presented in Section 4. The design of the requirements-based algorithm is discussed in Section 5. The results of the experiments that were performed to test the validity of the shared control system and to analyze the interaction characteristics between the machine and the different users are presented in Section 6. Finally, Section 7 presents the conclusions.

## 2. Related Works

**Figure 1:**
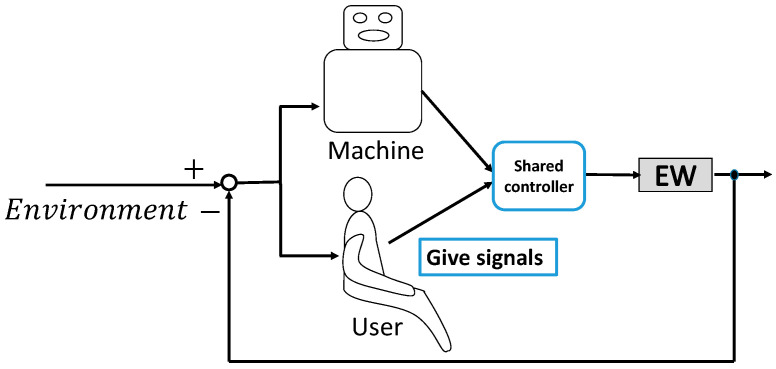
Construction of a shared control system for electric wheelchairs (EWs).

The construction of a shared control for EWs is shown in Figure 1. According to the definition of shared control, the user first expresses their intention through signals such as the movement of a joystick. A shared controller is then designed to allow the user and the machine to work together to operate an EW to the intended destination. Therefore, the challenge was how to design the machine and the shared controller.

Many types of shared control systems have been developed for EWs. Essentially, they are divided into three categories based on how users participate in the driving process [11,12]: The first category corresponds to behavior-based shared control systems, which are typically designed for individuals with disabilities who maintain a constant and significant operating ability. These systems only intervene in a few pre-defined situations, such as avoiding an obstacle or following a wall [13]. The key issue of this approach is how to execute the behavior. Many EWs are coupled with methods such as the dynamic window approach (DWA) [14] or the vector field histogram (VFH) [15], which are used when defined situations are detected or triggered by users. The second approach corresponds to goal-based shared control systems [16]. These systems first estimate a potential goal or sub-goal from the input signal of the user. Subsequently, several possible paths are generated by global and local planners, and the most feasible driving path is selected based on the input of the user. Although this system takes into account the intentions of the user, the EWs simply select the prepared path calculated by the planners, thereby significantly limiting the control authority of the user. The third approach corresponds to continuous shared control systems [6]. With these types of systems, user performance is evaluated online via predefined cost functions, and the control authority of the user is then determined by online evaluation results. However, driving an EW is a complicated, long-term process, where operation is different even under the same conditions. Real-time optimization limits user performance and the construction of cost functions is always difficult.

This study focused on designing a shared control system that considered the physical functions of users while maintaining their driving motivation. To make full use of the physical functions of users, this shared control system needed to fully understand the operating characteristics of the users in different driving situations. Additionally, driving motivation also needed to be fully considered to encourage users to actively participate in the EW driving process.

## 3. System Requirements Based on Target User

### 3.1. Operating Characteristics of Individuals with Severe Physical Impairments

**Figure 2:**
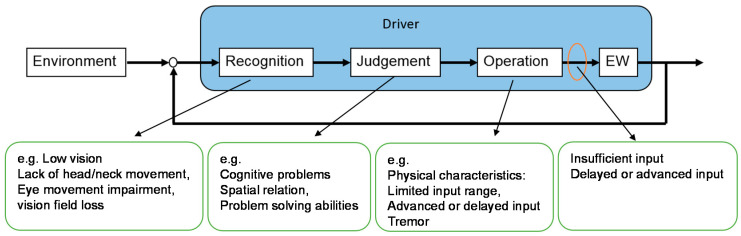
Driver inner model.

Many types of input devices have been developed for individuals with severe physical impairments, such as those using user-generated sound signals and electromyography signals. However, one of the purposes of this study was to make better use of the residual physical functions of users; therefore, this study decided on the use of continuous input devices, such as joysticks, for the target users. Previous research showed that although some input devices have been developed considering their physical functions, they still have difficulties operating such EWs, especially in situations where fine steering abilities are necessary [17,18]. Figure 2 shows a driver inner model. The driver first recognizes the environmental information and EW movements, then makes a judgment based on the recognition and gives operations based on the judgment results. Those who have difficulty driving an EW have one or more problems in the recognition process. As shown in Figure 2, visual impairment usually affects the recognition process. It usually manifests itself as low vision, low range of head/neck movement, impaired eye movement, or visual field loss [19]. Individuals with such problems cannot accurately perceive the position of obstacles. The ability to judge spatial relationships and the ability to solve problems are important for operating an EW [20]. Therefore, individuals with these cognitive problems cannot make accurate judgments. However, even with good recognition and judgment, the input signals of individuals with weak physical functions may be insufficient or excessive [17,18]. Problems in all three processes result in the input signals given by these users always being insufficient, advanced, or delayed.

The target users of this study are individuals with difficulties driving EWs. Their input characteristics are summarized as follows:

1. Limited input range. Individuals with extremely weak physical functions usually lack appropriate input devices. Although some devices have been developed with their physical functions in mind, during the development of their input devices, the user’s physical functions continue diminishing; thus, they cannot properly operate the designed devices after development.
2. Delayed or advanced input signals. For individuals with the problems discussed above, their input only intuitively represents the intention of the subjects, although driving an EW in real environments corresponds to complicated behavior. More accurate and frequent input is necessary, especially for situations where fine steering skills are required, which is almost impossible for them.

### 3.2. Driving Environment Settings

The living environment of individuals with severe physical impairments is extremely limited. Most of them move between their bedrooms, living rooms, and bathrooms [21]. Therefore, going straight and turning are the two most basic and important movements to complete these driving tasks. In this study, driving straight and turning left was used as the driving course, because we regarded turning left and right as an equivalent and symmetrical challenge.

### 3.3. System Requirements

The purpose of driving an EW is to allow users to reach their destinations; therefore, the system must be able to accomplish this goal. It is also necessary to make the driving process gradually safer and more comfortable, because safety and comfort are the two most important factors for EW driving [11]. As discussed above, the characteristics of target users and driving environments vary; therefore, the shared control system should be able to adapt to various types of users and driving environments. The objective of this study was to design a shared control system for individuals with weak physical functions by considering their residual physical functions and driving motivation. It is important to use their operating signals to the greatest extent possible to maintain their residual physical functions. Therefore, to utilize the operating capacity of users completely, it was necessary to develop a novel shared control method that assists by considering the entire driving process of an EW. Given the requirements of the EW driving process, the physical functions of users, and the input characteristics as discussed above, the requirements of the shared control system are summarized as follows:

1. The system should assist users in reaching their destination.
2. The complete driving process should be safe.
3. The complete driving process should be comfortable.
4. The system should gradually adapt to different users.
5. The system should gradually adapt to different environments.
6. The system should completely utilize the residual physical functions of individuals with disabilities.
7. The system should consider the driving motivation of users.

## 4. Construction of the Shared Control System

### 4.1. Modeling an EW

**Figure 3:**
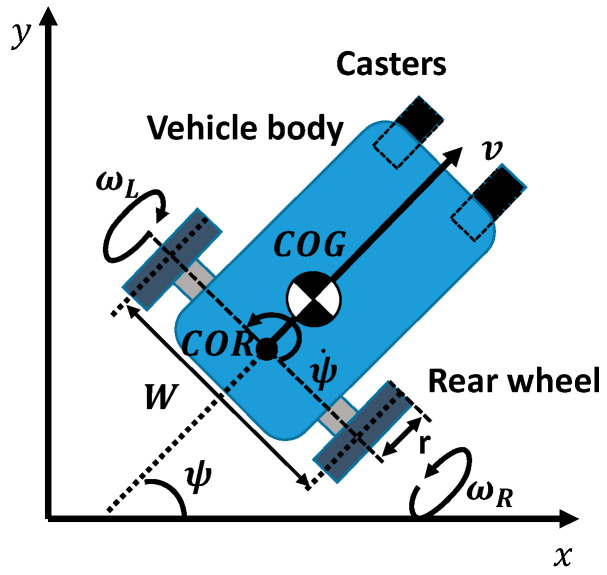
Top view of the proposed EW model.

Figure 3 shows the top view of the proposed EW. The EW is driven by two rear wheels, and two casters are used as the front wheels. The meanings of the parameters are listed in Table 1. The two assumptions are set as follows:

1. The slip between the wheels and the road can be neglected.
2. The EW will not move in the pitch direction, which means that the casters will not leave the road.

The straight and rotational motions of an EW can be expressed as (1) and (2), respectively.

(1) $$v=\frac{r\left(\omega_{R}+\omega_{L}\right)}{2}$$

(2) $$\dot{\psi }=\frac{r\left(\omega_{R}-\omega_{L}\right)}{W}\;$$

### 4.2. Concept of the Shared Control System

**Figure 4:**
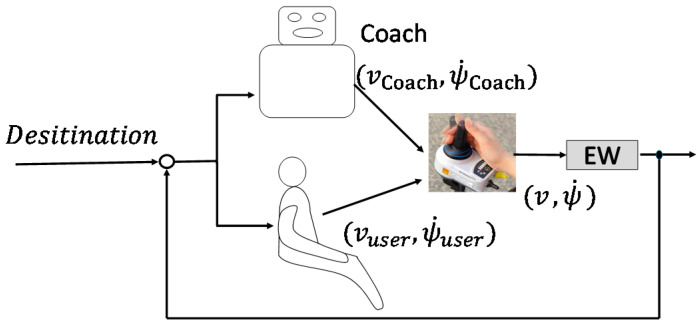
Shared control system concept.

Based on the requirements, the concept of the novel shared control system is shown in Figure 4. The user-machine relationship is similar to the relationship between a driving coach and a learner driver. This scheme is very similar to the continuous shared control, as discussed in previous research [6]. The difference is that, to make better use of the residual physical functions of users, the coach does not continuously correct the performance of users. The coach should intervene at an appropriate time depending on the performance of the learner driver. The coach should also be able to intervene in different ways depending on the different training purposes. For example, to use the residual physical functions of users, it was possible to decrease the intervention appropriately for individuals with error-correcting abilities.

### 4.3. Framework of the Shared Control System

**Figure 5:**
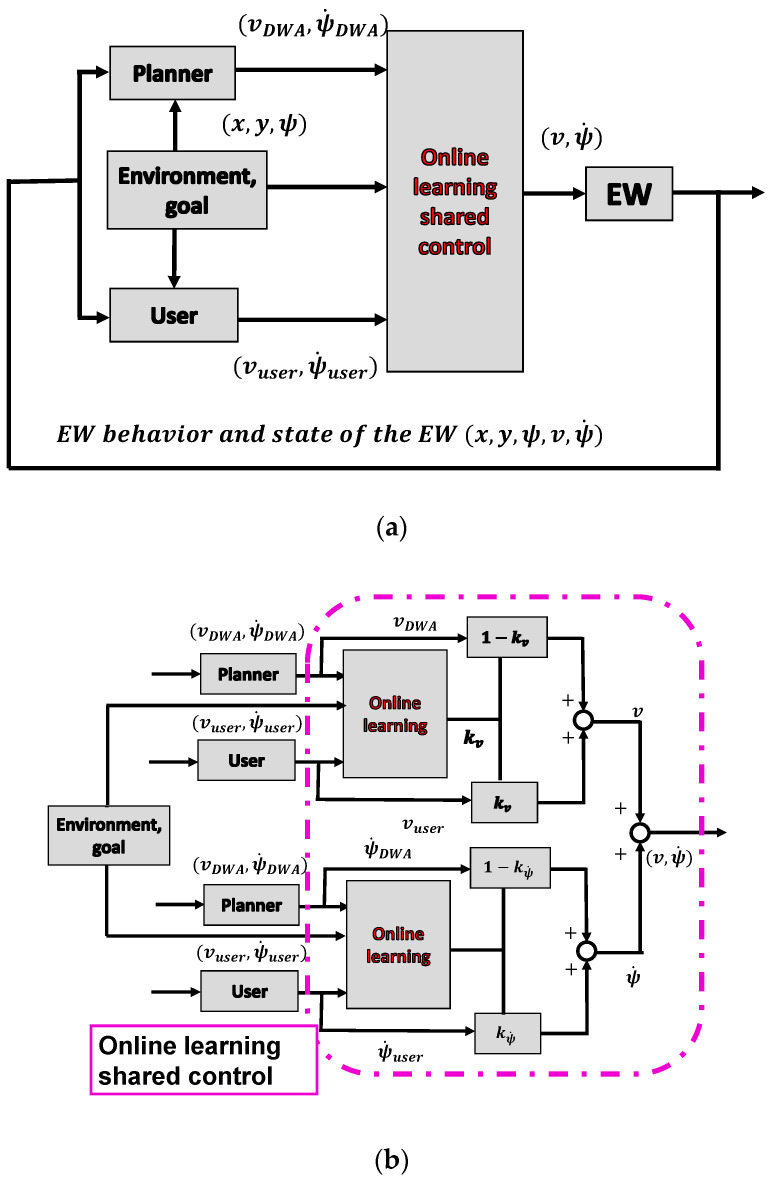
Construction of the shared control system: (**a**) framework of the shared control system and (**b**) online learning part.

To meet the requirements, the proposed shared control system (coach) was divided into two parts: the planner and online learning parts, as shown in Figure 5a. According to the analysis in the previous section, the coach has a standard in mind. When the operation of the user does not meet this standard, the coach will intervene appropriately. The planner part can be regarded as the operation standard of the coach, whereas the online learning part is the judge of the coach, to decide when and how to intervene. Users provided a velocity command ($v_{user},\;\dot{\psi_{user}}$) through some type of input device while operating the EW. Subsequently, user signals, planner velocity signals ($v_{Planner},\;\dot{\psi_{Planner}}$), and environmental information *(*x, y, ψ*)* were used in the online learning system. The control weight for the user was defined as $k_{v}$ and $k_{\dot{\psi }}$, whereas the control weight for the nonexpert planner was defined as $\left(1-k_{v},\;1-k_{\dot{\psi }}\right)$. The online learning part is shown in Figure 5b. For individuals with severe disabilities, the operating characteristics for going straight and turning are significantly different, and the straight and yaw motions of the EW were always analyzed separately [22]; thus, $k_{v}$ and $k_{\dot{\psi }}$ were calculated separately. The shared control equations are expressed as

(3) $$\{\begin{array}{c} v=k_{v}v_{user}+\left(1-k_{v}\right)v_{DWA}\\ \dot{\psi }=k_{\dot{\psi }}\dot{\psi_{user}}+\left(1-k_{\dot{\psi }}\right)\dot{\psi_{DWA}} \end{array}$$

## 5. Online Learning System Design for the Shared Control System

When a driving coach tries to teach a learner driver, the driving behavior is also observed, and the learner driver is only assisted when necessary. Therefore, the coach also needed to understand the behavior of the learner driver. Unlike teaching people how to drive a car, the people targeted in this study were individuals who have difficulties driving EWs. They cannot properly operate an EW, even if they received enough training. Therefore, it was important to ensure that both the user and coach understood each other as quickly as possible. The coach was also asked to completely use the operating ability of the users. In addition, driving an EW is a complicated, long-term process, where the operation is different even under the same conditions. Therefore, reinforcement learning (RL) was considered a suitable long-term optimization method for this shared control. This section discusses the definition and application of reinforcement learning in this study.

### 5.1. Definition of Reinforcement Learning

**Figure 6:**
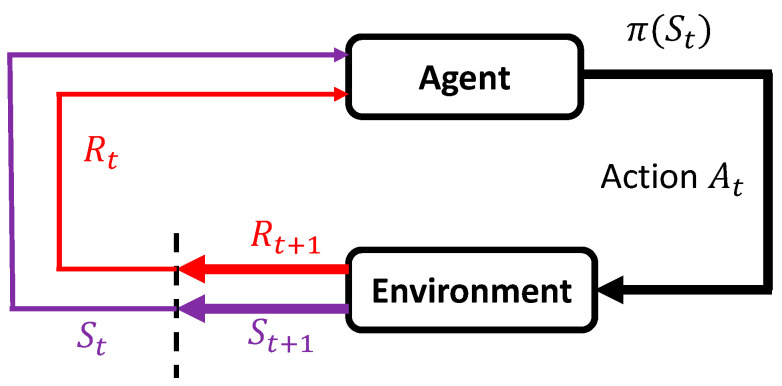
Structure of reinforcement learning.

Figure 6 shows the structure of RL [23]. The decision-maker is called the agent, and it interacts with the environment at each step. The agent acts, and the environment responds to the action and presents a new situation to the agent. Specifically, the agent interacts with the environment at a sequence of discrete time steps, *T* = [$t_{1},\;t_{2},\;t_{3}\ldots t_{n}$]. The environmental information received by the agent at each time (t) is defined as the state ($S_{t}$), and $\left(A_{t}\right)$ denotes the action sets that the agent possibly takes at this state. After taking the action, the agent receives the reward (R) and finds itself in a new state ($S_{t+1}$). How the agent chooses the action is called the agent policy, where $\pi_{t}(a|s)$ denotes the possibility that $A_{t}=a$ if $S_{t}=s\;$. The goal of RL is to obtain the optimal policy ($\pi^{\prime }$), maximizing the accumulated reward. Specifically, the optimal policy ($\pi^{\prime }\;\left(S\right)$) is represented in (4) by the state-action value function Q(S,a).

(4) $$\pi^{\prime }=\arg\underset{a\in A}{\max}Q^{\pi }\left(S,a\right)$$

### 5.2. Reinforcement Learning for the Shared Control System to Determine the Control Weights

**Figure 7:**
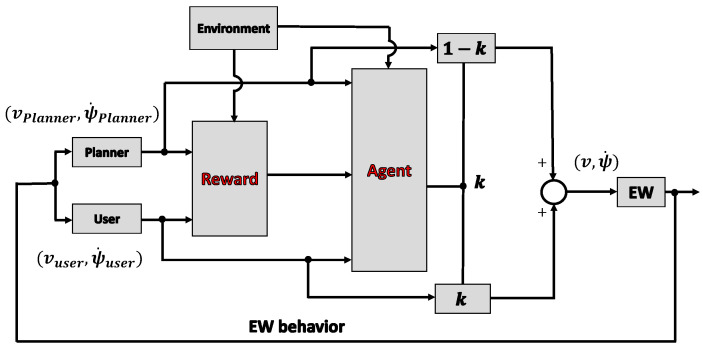
Structure of shared control using reinforcement learning.

The online learning part aims to assign user control weights in different environments, considering physical functions and driving motivation. As discussed above, reinforcement learning is a long-term optimization method that could be used to solve this problem by designing an appropriate reward (R) and state (S), and choosing a suitable reinforcement learning algorithm. The structure of the shared control system using reinforcement learning is shown in Figure 7. The reward (R) was first calculated using user input, planner input, and environmental information. Subsequently, the user and planner inputs, environmental information, and rewards (R) were used in the reinforcement learning algorithm to calculate the user control weights.

In reinforcement learning, the purpose of the agent is formalized using a scalar value reward (R) from the environment to the agent. In this research, the purpose of the agent was to assist the users in completing the driving task by considering their physical functions and maintaining their driving motivation. In other words, the reward (R) should consist of information regarding the evaluation of the driving process, such as reaching the goal, safety, comfort, adaptation to users and environments, use of physical functions, and maintenance of driving motivation. This aspect of the study is discussed in Section 5.3.

Conversely, controlling a dynamic robot using reinforcement learning usually requires many pre-training trials. However, the target users were individuals with weak physical functions who have difficulties driving EWs; therefore, too many training trials could fatigue them. In addition, individuals with extremely weak physical functions could have their physical functions (operating ability) affected by many factors, such as the surrounding temperature and the holding position of the input device, resulting in different operating characteristics at different times. Therefore, this system had to be able to learn without pre-training and reach convergence within certain training trials. Proper design of states and action sets could solve this problem to some extent, which is discussed in Section 5.4.

### 5.3. Reward Design for the Shared Control System

As discussed above, the reward (R) should consist of information about the design requirements. However, it was first necessary to understand the EW driving process. Therefore, in this section, the driving characteristics are first investigated through experiments, and then the reward design method is proposed based on the investigation and design requirements.

#### 5.3.1. Driving Characteristics Investigation

To describe the characteristics of the drivers in more detail, we first asked several healthy people to drive an EW. The driving characteristics of the EW were then analyzed using the driving data. As discussed in Section 3, driving an EW in the setting of going straight and turning left was investigated here.

**Figure 8:**
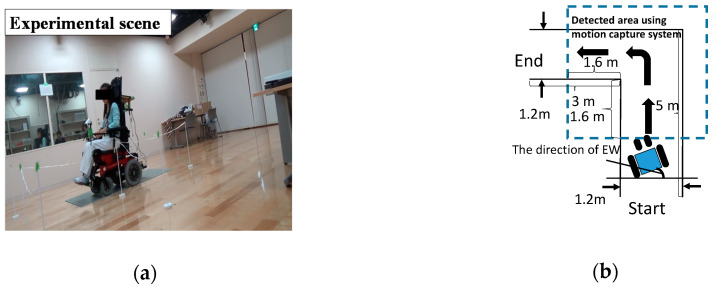
Experimental setup: (**a**) experimental scene and (**b**) course setting.

The EW used here was the EMC730, produced by the IMASEN Company. Four healthy young volunteers were asked to drive the EW. They were asked to practice driving for 5 min before the experiments, and each volunteer was asked to drive along the course 10 times during each experiment. The experimental setup is shown in Figure 8a. The course shown in Figure 8b was set up so that it was 1.2 m wide to meet the corridor width requirement (feet method). A motion capture system (Cortex, Motion Analysis Corporation, Santa Rosa, CA, USA) was used to record the EW trajectories and speeds. The input signals of the volunteers were also recorded.

**Figure 9:**
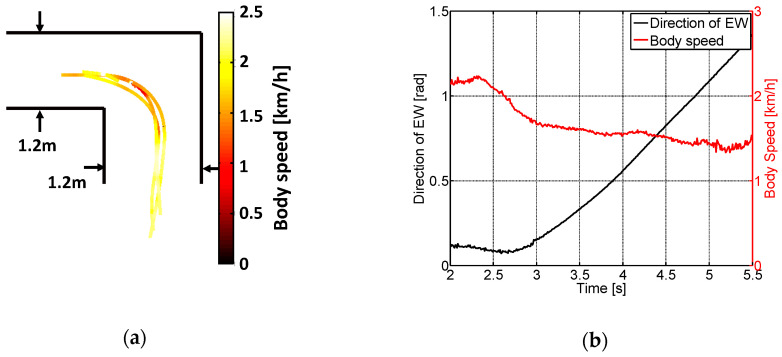
Experimental results of the driving characteristics: (**a**) trajectories and body speeds and (**b**) motion of an EW.

Figure 9a shows the EW trajectories and body speeds. Each line shows the trajectory of the midpoints of the rear wheels. Additionally, the color bar represents the body speed of the midpoint: the brighter the color, the faster the body speed. Figure 9b shows an example of body speed and EW direction. It can be seen that users tend to operate continuously when driving the EW going straight and turning left.

#### 5.3.2. Reward Design for the Shared Control System

To meet the design requirements, the reward for continuous driving was divided into four parts: current reward, predicted reward, participation reward, and task reward. The current reward was determined by the difference between the speed of the current moment and the previous moment, considering indicators such as comfort when driving an EW [11].

**Figure 10:**
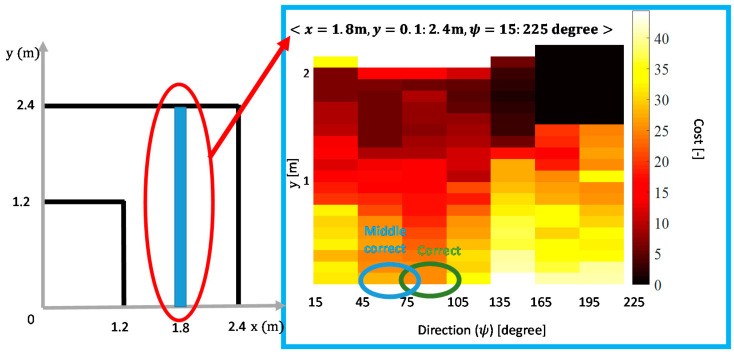
Example of cost determination using Hybrid A* (HA*) <*x* = 2 m, *y* = 0.1:2.4 m, *ψ* = 15:$225^{\circ }$>.

A Hybrid A* (HA*) [24] based method was designed to calculate the predicted reward based on whether the EW was driven to the goal. The HA* is a heuristic method that determines the cost from the current state to the goal state. The HA* method was initially used to calculate the cost of the current state. The difference between the cost calculated for the current moment and the next moment could be used as the predicted reward, assuming that the inputs of the user and the planner remained unchanged during one cycle. The items for the cost function of this HA* method are shown in Table 2, which were designed by considering the requirements and characteristics of individuals with severe disabilities. Here, $G_{n}$ denotes the positive gain for each cost item, *HC* denotes the heuristic cost using A*, *d* denotes the distance to the closest wall, α is the steering angle, and *CC* and *BC* are the direction change and backward penalty, respectively. Figure 10 shows an example for states $<x=2\;m,\;y=0.1:2.4\;m,\;\psi =15:225^{\circ }\;>$, where the color represents the cost for each state: as the cost increases, the color becomes lighter.

The participation reward was simply calculated by $P_{2}*k_{t-1}$, to evaluate whether the input of the user was used to drive the EW, where $k_{t-1}$ denotes the control weight of the user at the last moment and $P_{2}$ denotes the gain for this reward. A larger $P_{2}$ might make the algorithm more inclined to use the input of the user.

Finally, a task reward was set to evaluate whether the user completed the task. The system obtains a positive reward ($R_{goal}$) if the EW achieved the goal, a negative reward ($-R_{collide}$) if the EW collided, and zero task reward otherwise.

The entire reward function is shown in (5). The predicted reward corresponded to the difference between the cost of the state for the next moment and the current moment, and represented the evaluation of the current behavior of the EW. The current cost was calculated from the change in speed of the EW, as acceleration is the main factor affecting comfort. Participation rewards were used to evaluate whether a user actively participated in the driving process. Finally, the task reward aided the system in evaluating driving behavior throughout the entire driving process.

(5) $$\mathrm{Reward}=\underset{Predicted\;reward}{\underbrace{Cost_{current}-Cost_{next}}}+\underset{Current\;reward}{\underbrace{P_{1}*\mathrm{abs}\left(v_{t}-v_{t-1}\;\right)}}\;+\underset{Participation\;reward}{\underbrace{P_{2}*(1-k_{t-1})}}+\underset{Task\;reward}{\underbrace{R_{goal}-R_{collision}}}\;\;(P_{1},P_{2}<0),$$

### 5.4. States and Action Sets Design for the Shared Control System

The states of the entire shared control system were defined as <x, y, ψ>, where x, y denotes the position information and ψ represents the orientation of the EW. When discretizing the states, if the density of the states was too low, it could not accurately reflect the motion of the EW. In contrast, if the density of the states was too high, it could affect the convergence of the reinforcement learning algorithm. Therefore, it was important to design the resolution of the state to achieve the purpose of this application. To reduce the EW orientation classifications, we classified the EW orientation in the current state by considering the ultimate goal of EW driving rather than considering only the size of the orientation in Cartesian coordinates. The EW orientation (ψ) was classified into the following three categories: correct direction, middle correct direction, and wrong direction. In this application, the resolution of the orientation was set at $30^{\circ }$. As shown in Figure 10, concerning the position (x = 2 m, y = 0.1 m), ψ between $75^{\circ }\;$ and $105^{\circ }$, and $45^{\circ }$ and $75^{\circ }$ were considered the correct direction and middle correct direction, respectively, whereas ψ different from these ranges were considered the wrong direction.

The frequency of this system was set at 10 Hz. Given that the speed of an EW in indoor environments typically ranges from 1.5 to 2.5 km/h, the EW moved approximately 4–7 cm within a cycle. Position information was presented using grid cells that corresponded to squares of a certain length. We compared the different learning results by using grid lengths of 10, 20, and 30 cm through simulations. The results show that a rapid and accurate convergence was obtained after approximately 10 training trials when the grid size was 20 cm. In the case of 10 cm, the policy converged but required more than 30 training trials, and in the case of 30 cm, the policy did not converge even after more than 50 training trials. This was because the side length of the grid size was too large. The position of the EW could vary significantly when the EW passes through the grid under different conditions. Thus, the system could not accurately learn the correct policy for user control weight.

### 5.5. Sarsa Learning Based Shared Control Algorithm

Various methods have been developed to solve reinforcement learning problems (value function estimation), and can be classified into three types: dynamic programming-based optimal control approaches, the Monte Carlo method, and temporal difference methods such as the Q-learning and Sarsa learning methods.

Sarsa learning is a widely used online learning algorithm, especially in the field of dynamic planning within unknown environments [25,26]. In this study, Sarsa learning was applied to calculate the optimal policy used to select the optimal user control weight in different states. The state sequence ($S_{t}$), action ($A_{t}$), reward function (R), action value function (Q), and optimal policy were designed in the aforementioned parts of this section. The step for updating the action-value function is shown in (6), where $\alpha_{t}$ denotes the learning factor and γ denotes the discount factor.

(6) $$Q\left(S_{t},A_{t}\right)=Q\left(S_{t},A_{t}\right)+\alpha_{t}\left[R_{t}+\gamma Q\left(S_{t+1},A_{t+1}\right)-Q\left(S_{t},A_{t}\right)\right],$$

Furthermore, we applied the softmax method to select the actions [23]. This is a method that varies the probabilities of the actions as a graded function of the estimated value. Additionally, we used the Boltzmann distribution to show the action selection probability of each given state. The Boltzmann distribution is shown in (7), commonly used in the action selection process in RL problems, where τ denotes a positive temperature parameter affecting the exploration/exploitation trade-off and $P_{A_{N}}$ denotes the possibility of action ($A_{N}$).

(7) $$p_{A_{N}}=\frac{e^{Q\left(A_{N}\right)/\tau }}{\sum_{i=1}^{n}e^{Q\left(A_{i}\right)/\tau }},$$

The complete algorithm is shown in Table 3. The system obtained a set of data $\left\{S_{t},A_{t},R_{t}\right\}$ after each update.

The stop condition of the training process was set as follows:

1. *Q* (*s*,*a*) for each state only changes to a certain value.
2. The rank *Q* (*s*,*a*) for each state only changes to a certain value.
3. The latest trajectory does not include new states.
4. Consecutive successes of the above condition.
5. The number of training trials exceeds a certain number.

## 6. Experiments: System Effectiveness and User-Machine Interactions

There were two purposes for these experiments. The first was to verify the effectiveness of the shared controller with real users. The second was to analyze the characteristics of user-machine interactions. The experiments were first carried out in a virtual reality (VR) platform, as this system requires several training trials and for safety reasons.

### 6.1. Experimental Setup

**Figure 11:**
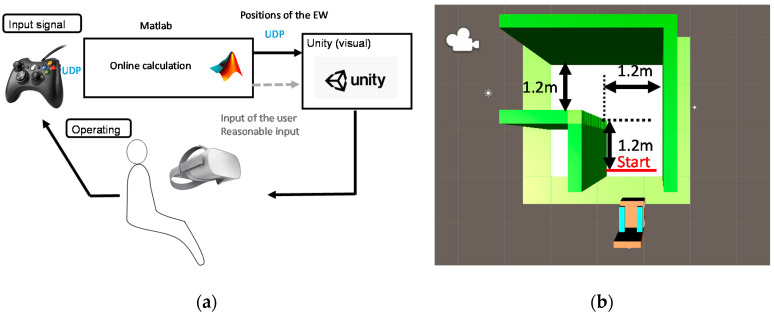
(**a**) Experimental setup and (**b**) experimental scene.

The experimental setup is shown in Figure 11a. The user provided the operating signals through a joystick and the input signals were then transmitted to MATLAB through the user datagram protocol (UDP). MATLAB was used to calculate the real-time positions and orientations using information about the environment and the input signals. Subsequently, the calculation results were sent to Unity (Unity Technologies) through the UDP. Users could train their driving ability in the virtual reality world through a head-mounted virtual reality display (Oculus). The sampling frequency of this shared control system was set as 10 Hz. Figure 11b shows the experimental scene, where the course was set at 1.2 m width, as discussed in Section 5.3.

**Figure 12:**
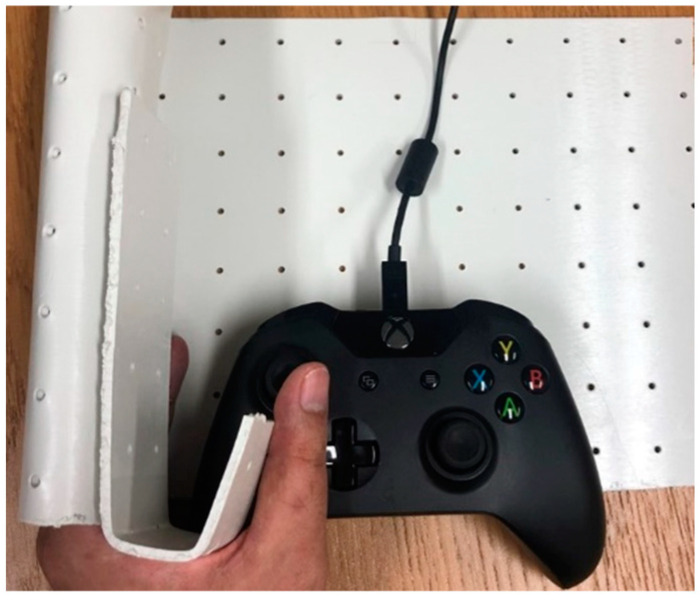
Use of a restraining device to reproduce the insufficient input scenario.

**Figure 13:**
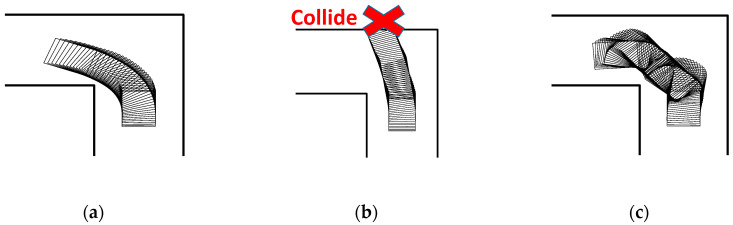
Driving examples with different input characteristics: (**a**) normal user, (**b**) user with insufficient input, and (**c**) user with oversteered input.

As discussed in Section 3, there were two input characteristics for individuals who have difficulties operating an EW: insufficient input and oversteered (excessive) input. Ten volunteers participated in the experiments. In the case of insufficient input, the volunteers were required to use a restraining device to limit the leftward movement of the thumb. As shown in Figure 12, the restraining device was made of polyform splinting material (SAKAIMED Company, Tokyo, Japan), and the shape of the material could be changed by heating. The most important reason for the oversteering phenomenon is that the user provides more steering signals than expected, causing new errors, and this vicious cycle leads to the oversteering phenomenon of the entire driving process. To achieve the oversteering effect, the following algorithm was added to deal with the input from the user: If the input for changing direction (more than 30% of the maximum) lasted more than 0.3 s, the input gain would be increased three times. Figure 13 shows examples of normal users, users with insufficient input (with the restraining device), and users with oversteered input (by changing the input gain), showing that the auxiliary settings used in this section to reproduce the input characteristics were correct. It should be noted here that all volunteers were required to understand their input characteristics by operating without the shared control system 5 times before starting the experiments.

### 6.2. Experimental Method

Two experiments were conducted with each participant to verify the effectiveness of the shared control system and analyze the user-machine interactions. Before each experiment, each participant was asked to reproduce the insufficient and oversteered inputs using the methods introduced in Section 6.1 to understand their input characteristics. After each experiment, a questionnaire survey was conducted. The content of the questionnaire is shown below:

1. The control is easy.
2. The control requires concentration.
3. The control causes physical fatigue.
4. The training duration is long.
5. The EW is controlled by the user.
6. The control is mostly satisfactory.

Subjects were asked to complete the experiments and answer a questionnaire based on a Likert scale (i.e., 1 = strongly disagree, 2 = disagree, 3 = neither agree nor disagree, 4 = agree, 5 = strongly agree) [27]. The participants were also asked to answer the Driving Behavior Questionnaire (DBQ) [28] and the Driving Style Questionnaire (DSQ) [29], which were used to analyze the driving characteristics of the users.

### 6.3. Experimental Results

The objective results mainly showed the effectiveness of the shared control system and how the shared control system assisted and interacted with the users, whereas the subjective feelings of the users about the shared control system are shown in the subjective results.

#### 6.3.1. Objective Results

**Figure 14:**
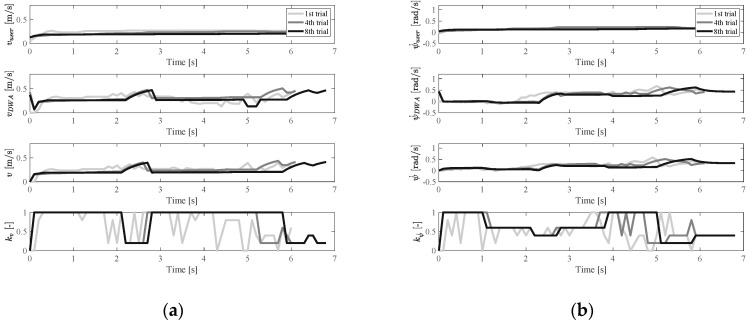
Velocity and control weight change in the insufficient case: (**a**) $v_{user},\;v_{DWA},\;v,\;\mathrm{and}\;k_{v}$ change and (**b**) ${\dot{\psi }}_{user},\;{\dot{\psi }}_{DWA},\;\dot{\psi },\;\mathrm{and}\;k_{\dot{\psi }}$ change.

Figure 14 shows an example of the insufficient case, where the participant can hardly give a $\dot{\psi }$ signal with the restraining device. Taking the 7th training trial (the last training trial) as an example, the planner began to dominate the control of $\dot{\psi }$ after 2.7 s. It was found that the user could still take most of the control in the v direction, because during the 2.7–4.7 s range, the user input ($v_{user}$) and DWA input ($v_{DWA}$) were very similar.

**Figure 15:**
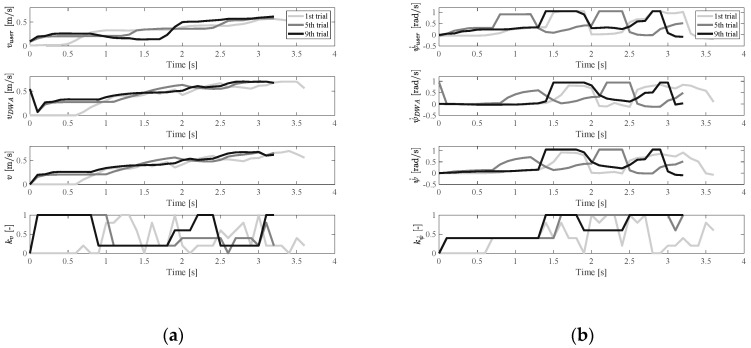
Velocity and control weight change in the oversteer case (active user): (**a**) $v_{user},\;v_{DWA},\;v,\;\mathrm{and}\;k_{v}$ change, (**b**) ${\dot{\psi }}_{user},\;{\dot{\psi }}_{DWA},\;\dot{\psi },\;\mathrm{and}\;k_{\dot{\psi }}$ change.

**Figure 16:**
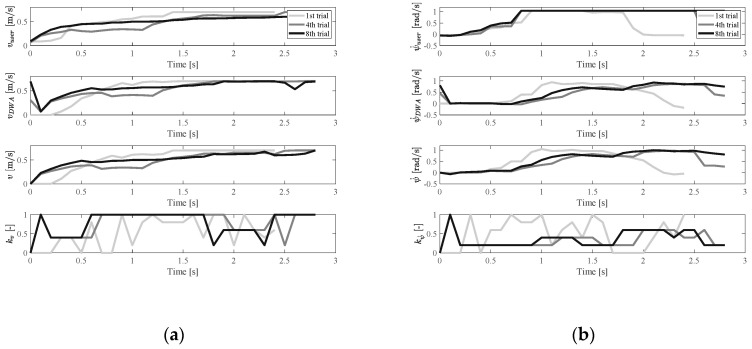
Velocity and control weight change in the oversteer case (passive user): (**a**) $v_{user},\;v_{DWA},\;v,\;\mathrm{and}\;k_{v}$ change, (**b**) ${\dot{\psi }}_{user},\;{\dot{\psi }}_{DWA},\;\dot{\psi },\;\mathrm{and}\;k_{\dot{\psi }}$ change.

Figure 15 and Figure 16 show the results of users with oversteered input characteristics. It was found that, besides the physical functions (operating characteristics), the user’s personality also affected the results of the experiments. Users with oversteered input characteristics could be divided into two categories based on how users adapted to the controller. The first category corresponds to active users, who were positively involved in the training process. An example of experimental results for an active user is shown in Figure 15. The second category corresponds to passive users. The passive users tended to rely on the fact that the shared controller gradually helped them to complete the driving, and they maintained the same operating pattern during training. An example of a passive user is shown in Figure 16. The pattern of their input signals remained unchanged, making the shared EW control more similar to autonomous driving. Of the ten subjects, seven were active users and three of them were passive users.

#### 6.3.2. Subjective Results

**Figure 17:**
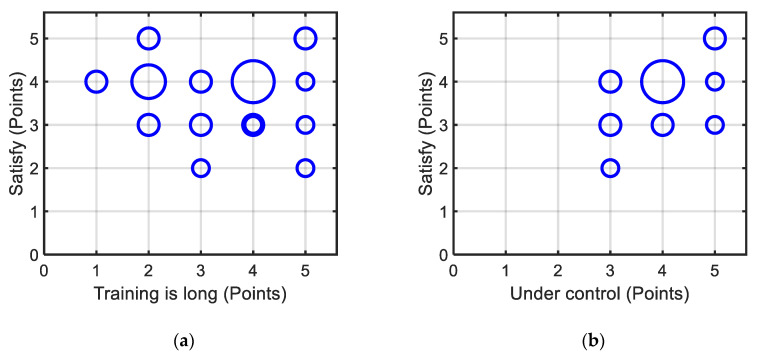
Results of the questionnaire. The larger the circle, the greater the number of users in that situation: (**a**) relationship between training duration and overall satisfaction, and (**b**) relationship between the feeling of under control and overall satisfaction.

As almost all training sessions did not exceed 3 min, the shared system did not cause user fatigue or demand too much attention, and users also found the training time acceptable. However, if the training trials exceeded 15 times, users often felt that the training duration was too long. As shown in Figure 17a, the points for the sixth item (the control is mostly satisfactory) tended to be high if the points for the fourth item (the training duration is long) were low. Figure 17b shows the relationship between the fifth item (the EW is controlled by the user) and the sixth item (the control is mostly satisfactory). Some passive users also thought that the EW was under their control; this may be because they thought that they had already understood the assistance method of the shared control system and the driving process was within their imagination. From the personality tests (DBQ and DSQ), it was found that those who defined themselves as defensive drivers were active users.

### 6.4. Discussion

**Figure 18:**
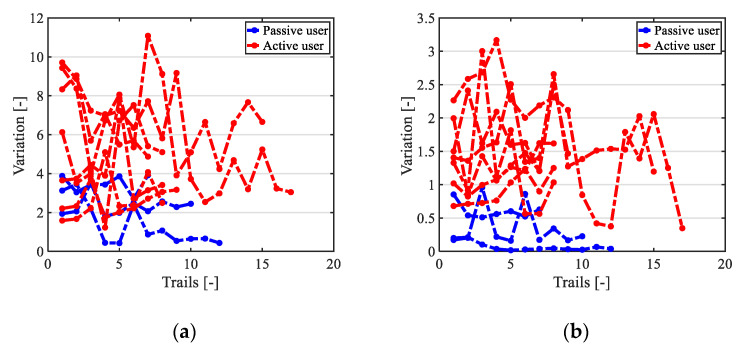
Input changes of active and passive users: (**a**) input changes in v direction and (**b**) input change in $\dot{\psi }$ direction.

The experimental results first showed the effectiveness of the proposed shared control system, which could be used by users with different input characteristics. The shared control system was found to help users make a safe left turn by increasing at least one of the DWA control weights. According to Section 5.3.2, gains in the reward function (5) might have a large influence on user-machine interactions. Therefore, it could be inferred that adequately improving one of the participation gains (e.g., a $P_{2}$ increase in $\dot{\psi }$) might give users more opportunities to train their steering control ability. The input characteristics of passive and active users were also very different. Figure 18 shows the input changes of different users, where the red and blue lines represent the active and passive users, respectively. The variation was calculated using (8), which represents the average input change in the v and $\dot{\psi }$ directions, where *t* and △t denote the time and the sampling time, *n* denotes the total sampling number, and *Input*(*t*) denotes the relative input signal at time *t*. It was found that active users were more inclined to change their operations, especially in the $\dot{\psi }$ direction. Based on the above discussion, the operating ability to control v and $\dot{\psi }$ could be trained separately.

As discussed in Section 3, the residual physical functions of users varied, and a more rational choice of parameters might improve user satisfaction. In addition, it was possible to make those passive users more actively involved in the shared control system with some prior education and instructions.

(8) $$\mathrm{Variation}=\overset{n}{\underset{t=1}{\sum }}\left(\frac{\left|Input\left(t+1\right)-Input\left(t\right)\right|}{n△t}\right)$$

## 7. Conclusions

In this study, a novel shared control system was developed for individuals with difficulties in operating EWs. This novel shared control system focused on utilizing the residual physical functions of users and maintaining their driving motivation. The requirements of the shared control system were first obtained by analyzing the operating characteristics of the target users and their lifestyles. Then, a novel reinforcement learning-based shared control scheme was proposed based on the design requirements. The effectiveness of the shared control system was verified through VR-based experiments, in which the shared control system could be adapted to various types of users. The experimental results also showed that some users could not actively participate in the shared control system, as they tended to rely on adaptation from the machine side. For these users, it was important to provide some directions beforehand.

In the future, the parameters of the shared control system, such as reward gains, could be designed considering the operational ability of users and the training purpose. This paper only discussed individuals with severe physical impairments who still have some physical functions to operate the interfaces with, such as tiny joysticks. For those people who cannot use joystick-type controllers, the proposed system should be extended for more interfaces, such as sound and EMG signals. Considering different operating devices will lead to different driving characteristics, so the parameters for the shared control system and reward design will need to be modified accordingly.

## Figures and Tables

**Table 1:** Parameters of the EW model.

Parameters	Meaning of the Parameters
$\;\omega_{R},\omega_{L}$	Angular velocity of the right (left) wheel
r	Radius of the rear wheels
W	Width of the EW
$v,\dot{\psi }$	Velocity of the straight (yaw) motion
x,y	Position in world coordinate system
COG	Center of gravity
COR	Center of rotation

**Table 2:** Items for the cost function in HA* to calculate the predicted reward.

Cost Items	Calculating Method
Approaching the goal	$\;G_{1}*HC\;$
Safety	$\;G_{2}*d\;$
Magnitude of the input	$\;G_{3}*\alpha \;$
Input change	$\;G_{4}*\delta \alpha \;$
Forward/backward change	$\;G_{5}*CC\;$
Backward	$\;G_{6}*BC\;$

**Table 3:** Shared control algorithm for EW driving.

Algorithm: Sarsa Learning-Based Shared Control Algorithm
1. $t=0,\;\;\gamma =0.8,\;\;\alpha_{t}=0.05,\;\;R_{t}$ calculated by reward function
2. Recursively compute until the stop condition is met
3. Recursively compute until reaching the goal
4. Obtain current state $S_{t}\;$
5. Decide action $A_{t}=\left(k_{v},k_{\dot{\psi }}\right)$ by Sarsa learning
6. Send $\left(k_{v},k_{\dot{\psi }}\right)$ to the system, calculate output $\left(v,\dot{\psi }\right)\;$
7. Send output $\left(v,\dot{\psi }\right)$ to the EW
8. Calculate the next state $S_{t+1}\;$
9. Update the Q-table, Load $R_{t}\;$
